# TLDc proteins: new players in the oxidative stress response and neurological disease

**DOI:** 10.1007/s00335-017-9706-7

**Published:** 2017-07-13

**Authors:** Mattéa J. Finelli, Peter L. Oliver

**Affiliations:** Department of Physiology, Anatomy and Genetics, University of Oxford, Parks Road, Oxford, OX1 3PT UK

## Abstract

Oxidative stress (OS) arises from an imbalance in the cellular redox state, which can lead to intracellular damage and ultimately cell death. OS occurs as a result of normal ageing, but it is also implicated as a common etiological factor in neurological disease; thus identifying novel proteins that modulate the OS response may facilitate the design of new therapeutic approaches applicable to many disorders. In this review, we describe the recent progress that has been made using a range of genetic approaches to understand a family of proteins that share the highly conserved TLDc domain. We highlight their shared ability to prevent OS-related cell death and their unique functional characteristics, as well as discussing their potential application as new neuroprotective factors. Furthermore, with an increasing number of pathogenic mutations leading to epilepsy and hearing loss being discovered in the TLDc protein TBC1D24, understanding the function of this family has important implications for a range of inherited neurological diseases.

## Introduction

The oxidative stress (OS) response is an essential cellular process that is required to maintain the physiological levels of reactive oxygen species (ROS), by-products of oxygen metabolism. ROS are essential intracellular messengers (Rhee 2006; Sauer et al. 2001), although any imbalance in the levels of ROS can cause damage to DNA, proteins and lipids, and may lead ultimately to cell death (Ryter et al. 2007). The brain has a high metabolic rate, utilising twenty percent of the body’s total energy consumption at rest (Magistretti pierre and Allaman 2015), yet it has a relatively poor antioxidant capacity (Katalinic et al. 2005); thus, it is unsurprising that the central nervous system (CNS) is particularly sensitive to OS (Friedman 2011). Markers of OS are observed consistently in brain regions and cell populations affected by neurodegenerative disease, for example in Alzheimer’s disease (AD), Parkinson’s disease (PD) or amyotrophic lateral sclerosis (ALS) (Andersen 2004; Shukla et al. 2011). However, it is still unclear whether disruption of the OS response is a direct cause of neurodegeneration in these disorders, or whether OS occurs as a result of neuronal dysfunction (Andersen 2004; Ramanan and Saykin 2013).

Despite this debate, it has been long hypothesised that antioxidants may be valuable therapeutic targets applicable to neurological disease (Moreira et al. 2010). Consequently, the efficacy of neuroprotective intervention using small molecules with antioxidant properties has been tested—with some success—in numerous rodent and primate models of AD, PD and ALS [reviewed in (Kamat et al. 2008)]. Translation of these antioxidant strategies in patients has been limited, however (Persson et al. 2014). An alternative but related therapeutic strategy has since been proposed, whereby the endogenous antioxidant mechanisms are harnessed to provide more biologically targeted protection in the CNS (Chan and Chan 2015). Consequently, it is critical to identify and characterise novel antioxidant proteins that are able to counteract damaging ROS in the brain. In this review, we will discuss recent evidence from genetic and molecular studies that the Tre2/Bub2/Cdc16 (TBC), lysin motif (LysM), domain catalytic (TLDc) domain-containing proteins are important players in the OS response and are therefore potential therapeutic targets applicable to many neurological and neurodegenerative disorders.

**Fig. 1:**
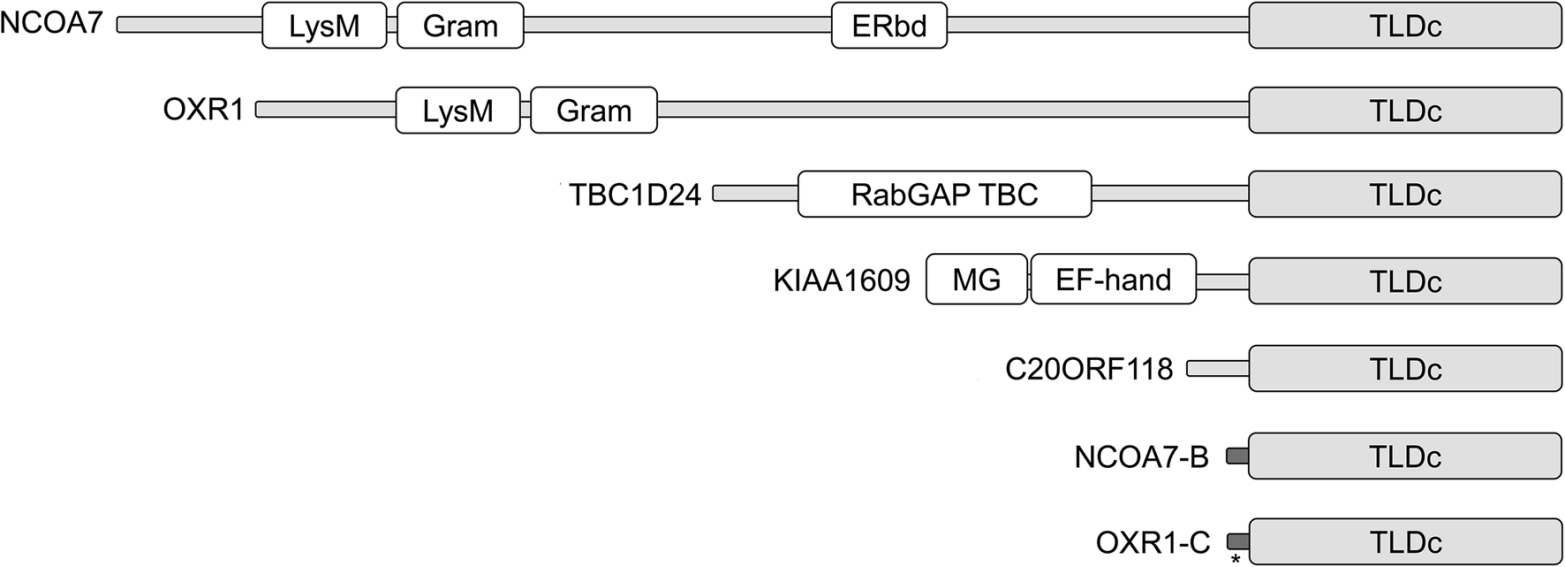
Domain architecture of mammalian TLDc proteins. Domains marked as TLDc: Tre2/Bub2/Cdc16 (TBC), Lysin Motif (LysM), Domain Catalytic; LysM: lysin motif; GRAM: GRAM domain; ERbd: estrogen receptor binding domain; RabGAP: GTPase activating protein; EF-hand: EF-hand domain; N-M: N-myristoylation domain. The accession numbers corresponding to the human isoforms as shown are: OXR1 (NP_001185461), OXR1-C (NP_001185464), NCOA7 (NP_001186548), NCOA7-B (NP_001186551), TBC1D24 (NP_001186036), KIAA1609 (NP_065998) and C20ORF118 (NP_001291712). The unique first coding exons of NCOA7-B and OXR1-C are shown in dark grey and the position of a premature stop codon mutation identified in OXR1-C in specific language impairment is marked with an asterisk (Chen et al. 2017)

The TLDc domain is a highly conserved protein motif present in several mammal proteins that share a protective function against OS: nuclear receptor coactivator 7 (NCOA7 or TLDC4), oxidation resistance 1 (OXR1 or TLDC3), TBC1 domain family member 24 (TBC1D24 or TLDC6), TBC/LysM-associated domain containing 1 (KIAA1609 or TLDC1) and TBC/LysM-associated domain containing 2 (C20ORF118 or TLDC2) (Fig. 1) (Finelli et al. 2016). The most evolutionary distant protein, TLDC5, or interferon-induced protein 44 (IFI44; protein accession number NP_006408), contains a putative TLDc domain with only 15% amino acid identity to the other members. Evidence that the TLDc domain is essential to normal human brain development and function has been highlighted recently by the identification of mutations in the TLDc domain of TBC1D24 in patients with myoclonic, focal or generalized epilepsy, DOORS (deafness, onychodystrophy, osteodystrophy, mental retardation and seizures) syndrome and lethal early-onset epileptic encephalopathy (Balestrini et al. 2016; Campeau et al. 2014a, b; Mucha et al. 2015). Therefore, understanding the molecular mechanisms that underlie the function of the TLDc domain will shed light on the OS response and its involvement in a wide range of neurological diseases.

## Oxidation resistance gene 1 (OXR1)

### OXR1 is protective against oxidative stress

OXR1 was first identified in a human cDNA library screen for genes that were able to rescue the DNA oxidation repair-defective phenotype of a spontaneous *E.coli* mutant (Volkert et al. 2000). This apparent OS-related function of OXR1 was shown to be evolutionary conserved in eukaryotes: the yeast strain *S. cerevisiae* with deletion of the homologue *scOxr1* was more sensitive to hydrogen peroxide (H_2_O_2_) treatment than controls (Volkert et al. 2000), and this phenotype could be rescued by expressing human OXR1 targeted to the mitochondria (Elliott and Volkert 2004).

Oxr1 has also been shown to be essential for CNS function in mammals. *Bella* mouse mutants, which harbour a homozygous spontaneous deletion spanning the entire coding sequence of *Oxr1*, display degeneration in the cerebellar granule cell layer from postnatal day (P)18, followed by rapidly progressive ataxia and death by P24 (Oliver et al. 2011). This dramatic phenotype was fully rescued by ubiquitous expression of an *Oxr1* transgene, confirming that the phenotype of the *bella* mutant is caused by loss of the gene (Oliver et al. 2011). Significantly, a new insertional mouse mutant in which the last 101 amino acids of the TLDc domain of Oxr1 is removed has an essentially identical phenotype to the *bella* Oxr1 deletion mouse, demonstrating the importance of the TLDc domain for the function of the protein (Finelli et al. 2016; Oliver et al. 2011). These particular findings are in line with in vitro studies demonstrating that the shortest Oxr1 isoform, containing almost exclusively the TLDc domain (Fig. 1), is as potent an antioxidant as the full-length protein (Finelli et al. 2016; Oliver et al. 2011).

Consistent with the original findings in bacteria that over-expressing *OXR1* is protective against OS, an increase in OXR1 levels has a similar effect in a wide range of mammalian cell types (Finelli et al. 2016; Murray et al. 2013; Yang et al. 2014). Furthermore, this protective function has been established in other genetic systems. For example, *Drosophila* embryos reared on an OS-inducing H_2_O_2_ solution are more likely to survive as adults when injected with Oxr1 from the silk moth *Bombyx mori* (bm), and *bmOxr1* also significantly increases the 50% survival time of *Drosophila* reared on a control diet by 11% (Kobayashi et al. 2014). Although this particular finding has not been replicated in other invertebrate models, these data suggest that Oxr1 is beneficial for survival, both under induced OS and physiological conditions.

To investigate whether Oxr1 could be similarly protective in a mammalian genetic model, a mouse over-expressing Oxr1 specifically in neurons was crossed with the well-characterised superoxide dismutase 1 (hSOD1^G93A^) mouse model of ALS (Gurney et al. 1994; Liu et al. 2015). Increasing Oxr1 levels in hSOD1^G93A^ mice not only delayed the expression of OS-related markers in the spinal cord, but it also improved motor neuron survival, motor coordination and life-span (Liu et al. 2015). Interestingly, Oxr1 binds to two other proteins that are associated with familial forms of ALS, TAR DNA-binding protein 43 (TDP-43) and fused in sarcoma (FUS) (Finelli et al. 2015). In vitro co-expression of Oxr1 with TDP-43 and FUS showed that disease-causing mutations could influence the binding affinity to Oxr1, which in-turn was associated with a reduction in the aberrant cellular features associated with these mutants (Finelli et al. 2015). It was also observed that the intermediate TLDc-domain-containing OXR1 isoforms were up-regulated compared to age-matched controls in end-stage post-mortem spinal cord from patients with ALS, as well as a threefold increase in the full-length Oxr1 in spinal cords of pre-symptomatic hSOD1^G93A^ mice (Oliver et al. 2011). Together, these studies propose that OXR1 plays an important role in ALS-associated pathogenesis, and could even be a valuable therapeutic target for neurodegenerative disorders susceptible to OS.

### OXR1: functional hypotheses

It has been hypothesised that OXR1 might function by reacting directly with ROS (Finelli et al. 2016; Oliver et al. 2011; Sanada et al. 2014; Yang et al. 2014), similar to antioxidant enzymes such as SOD1 (McCord and Fridovich 1969) or glutathione reductase (Massey and Williams 1965). Indeed, over-expression of OXR1 can reduce superoxide (Finelli et al. 2016) or H_2_O_2_ levels in vitro (Yang et al. 2014); however, to date, there is no evidence for an enzymatic activity of OXR1 or the TLDc domain. In fact, the oxidation of reactive cysteine residues in OXR1 does not occur in the catalytic range (Oliver et al. 2011) and the protein does not demonstrate any catalase or superoxide dismutase activity (Sanada et al. 2014). Alternatively, OXR1 could influence OS response pathways indirectly by modulating the activity of effector proteins. Indeed, an in silico pathway analysis performed on Oxr1 binding partners identified by co-immunoprecipitation in neuronal cells suggested that Oxr1 interacts with proteins involved in mTOR and EIF2 pathways (Finelli et al. 2015), key regulatory elements of the OS response (Maiese et al. 2012; Ron and Harding 2007; Teske et al. 2011). Another possible functional role of OXR1 is a more direct influence on the activity of known cellular antioxidants. In the mosquito *Anopheles cambiae* (*A. cambiae*), silencing the homologue of OXR1 significantly decreases the expression of both catalase and glutathione peroxidase (Gpx) by 92 and 82%, respectively. Moreover, chemically inhibiting the JNK pathway in larval *Bombyx mori* reduced Oxr1 levels in this organism (Su et al. 2016). Similarly, silencing Jun N-terminal kinase (JNK), a gene normally activated by stress, leads to a decrease in *Oxr1* expression in mosquitoes (Jaramillo-Gutierrez et al. 2010). This suggests a detoxifying cascade arising from JNK, regulating OXR1 transcription, which in turn induces expression of antioxidant enzymes (Jaramillo-Gutierrez et al. 2010, Su et al. 2016); thus, OXR1 may act downstream of the JNK pathway to regulate the OS response. Interestingly, a study describing the interactome of the proton V-ATPase pump identified Oxr1 and Ncoa7 as potential binding partners in the kidney (Merkulova et al. 2015). A functional link between vacuolar acidification and OS has been demonstrated in yeast (Milgrom et al. 2007); this would suggest another potential mechanism of regulation of OS by OXR1 through modulation of V-ATPase activity or stability of the V-ATPase complex.

Next, to dissect the pathway(s) downstream of OXR1 in more detail, transcriptomic analysis was carried out in the human cervical cancer (HeLa) cell line in which the level of OXR1 was knocked-down by 85% (Yang et al. 2015, 2014). More than 600 genes were differentially expressed in untreated OXR1-depleted cells compared to wild-type cells, with around half of these genes being similarly regulated in untreated or H_2_O_2_-treated conditions (Yang et al. 2015). Interestingly, the expression of several antioxidant genes, such as heme oxygenase (HO-1) and cyclin-dependent kinase inhibitor 1 (p21), was reduced in OXR1-depleted cells, both in untreated and treated conditions (Yang et al. 2015, 2014). Together with the data from knockdown studies in the mosquito, these studies suggest that Oxr1 can modulate, either directly or indirectly, the expression of ROS-reducing enzymes.

Gene ontology analysis of the differentially expressed genes in OXR1-depleted HeLa cells identified the p53 pathway as most significantly altered (Yang et al. 2015); these data therefore implicate OXR1 in cell-cycle progression and apoptosis via p53 and transcription of its target genes (Yang et al. 2015, 2014). However, given the importance of p53 in cancer cell physiology (Muller and Vousden 2014), it would be interesting to carry out a transcriptomic study in non-immortalised cell line to assess the relevance of this observation in a physiological context. At present, one can only speculate that OXR1 might modulate the transcription of p53-target genes by binding to p53 in the cytoplasm, modifying p53 activity or localisation, and facilitating the transcription of p53 target genes (Yang et al. 2015). This study also suggested a wider role for OXR1, in particular through the regulation of an array of essential transcription factors such as E2F transcription factor 8 (E2F8) and transcription factor 3 (TCF3) (Yang et al. 2015). However, how OXR1 would regulate the expression of these genes, both in non-treated and oxidative stress conditions, remains to be explored. There is some evidence for nucleolar and nuclear expression for OXR1 and the *Drosophila* OXR1 homologue *mustard (mtd*, see below) in vivo that would suggest a role in transcription (Fischer et al. 2001; Natoli et al. 2008; Wang et al. 2012). Indeed, NCOA7 has been shown to be nuclear localised and is implicated in transcriptional regulation (Arai et al. 2008; Durand et al. 2007; Paramanik and Thakur 2010; Shao et al. 2002); yet further studies are required to determine how OXR1 may influence transcription and whether this occurs by either a direct or indirect mechanism.

### OXR1 in infection and the immune response

In addition to its well-studied role in regulating OS sensitivity, OXR1 has been implicated in the response to bacterial infection. In a forward-genetic screen to identify host factors reducing proliferation of the parasite *Plasmodium gallinaceum*, a loss-of-function *Drosophila* mutant in the *l(3)82Fd* gene corresponding to the OXR1 homologue was identified, which presented a fivefold reduction in the number of mature oocytes of the parasite compared to the fly control line (Brandt et al. 2008). In addition, a gain-of-function mutant with an insertion in the same locus, renamed *mtd*, showed an increased tolerance to oral *Vibrio cholerae* infection mediated by the innate immune response (Wang et al. 2012). The mutant *mtd* phenotype could be mimicked by over-expressing a short *mtd* transcript containing only the TLDc domain, demonstrating that this region was important for sensitivity to infection (Wang et al. 2012). Consistent with this finding in *Drosophila*, knockdown of the *A. gambiae* OXR1 homologue resulted in a significant decrease in the *Plasmodium berghei* load in mosquitoes (Jaramillo-Gutierrez et al. 2009). As it has been observed previously that systemic ROS levels can influence *Plasmodium berghei* infection (Molina-Cruz et al. 2008), it was hypothesised that the lower rate of infection in *A. gambiae* following OXR1 silencing was due to the decrease in expression of ROS-clearing enzymes (Jaramillo-Gutierrez et al. 2010). However, the levels of ROS have not been assessed in this model system to confirm this hypothesis.

Supporting a potential role for OXR1 in the immune response in mammalian systems, mouse strains resistant to inflammation of the kidneys express significantly higher levels of Oxr1 than more sensitive strains (Li 2014). Subsequently, OXR1 was tested as a therapeutic target in a mouse model for an autoimmune disease caused by systemic lupus erythematosus (Li 2014). Mesenchymal stem cells (MSC) expressing OXR1 were delivered intravenously to these mice, leading to successful implantation in their kidneys. This resulted in the reduction of abnormal serum and urine nitric oxide levels, inhibition of cytokine and interleukin up-regulation, and a decrease in T-lymphocyte and macrophage infiltration into the kidneys (Li 2014). Somewhat related results were obtained in the hSOD1^G93A^ ALS model, where a reduction in the expression of several neuroinflammatory and complement activating markers occurred in SOD1 mutants over-expressing Oxr1 (Liu et al. 2015); this in turn led to decreased astrogliosis and microgliosis in spinal cord of hSOD1^G93A^ mice (Liu et al. 2015). Given the established links between inflammation, complement activation and neurodegeneration (Cappellano et al. 2013), it will be essential to investigate further how OXR1 is able to modulate the immune response and if this is a key feature of its neuroprotective properties.

## TBC1 domain family member 24 (TBC1D24)

### TBC1D24 is mutated in a range of human genetic disorders of the nervous system

The importance of TBC1D24 in normal brain function was first demonstrated by the identification of mutations in patients with various forms of epilepsy (Corbett et al. 2010; Falace et al. 2010). Indeed, this finding was the first genetic cause of human epilepsy to be discovered by exome sequencing. Since then, over 30 combinations of compound heterozygous or homozygous mutations in *TBC1D24* have been reported, spanning almost the entire coding sequence (Campeau 2016). These mutations cause a surprisingly broad range of disorders, including familial infantile myoclonic epilepsy (Falace et al. 2010; Poulat et al. 2015), early onset progressive encephalopathy associated with myoclonic epilepsy with severe cerebral neurodegeneration (Guven and Tolun 2013), familial malignant migrating partial seizures of infancy (Milh et al. 2013), early infantile epileptic encephalopathy (Stražišar 2015), lethal neonatal seizure disorder (Lozano et al. 2016), focal epilepsy associated with intellectual disability (Corbett et al. 2010), multifocal myoclonus with cerebellar dysfunction (Doummar 2015), focal epilepsy associated with developmental delays and head growth deceleration (Appavu et al. 2016). Most recently the disease spectrum has expanded to include multifocal polymyoclonus with neurodevelopmental delay (Ngoh et al. 2017) and alternating hemiplegia with recurrent episodes of *epilepsia partialis continua* (Ragona et al. 2017). Mutations in TBC1D24 are also associated with DOORS syndrome, a rare disorder characterised by hearing loss and shortened terminal phalanges with small nails of the hands and feet, in addition to seizures and neurodevelopmental delay (Campeau et al. 2014b). TBC1D24 mutations have been identified in approximately half of those diagnosed with DOORS syndrome to date; thus, there are likely to be other causative genetic factors as well as potential clinical overlap with disorders such as Coffin-Siris syndrome that share many, if not all, of the cardinal features of DOORS (Campeau et al. 2014a, b). This disease association is particularly interesting as patients with compound heterozygous TBC1D24 mutations can also present with hearing loss associated with an early-onset epileptic encephalopathy (Stražišar 2015); furthermore, dominant (Azaiez et al. 2014; Zhang et al. 2014) and recessive (Rehman Atteeq et al. 2014) TBC1D24 mutations can also cause non-syndromic hearing impairment, with no reported epileptic phenotype (Azaiez et al. 2014; Rehman Atteeq et al. 2014; Zhang et al. 2014). Consistent with the range of pathologies described in patients carrying TBC1D24 mutations, *Tbc1d24* is expressed in the early embryonic brain as well as in the inner and outer hair cells and in the spiral ganglion neurons in rodents (Falace et al. 2010; Finelli et al. 2016; Rehman Atteeq et al. 2014; Zhang et al. 2014).

By compiling the epilepsy and DOORS-associated TBC1D24 mutations detected to date with their corresponding clinical features, there is some evidence that the more severe clinical phenotypes are associated with nonsense, frameshift or splice-site mutations (Balestrini et al. 2016). TBC1D24 protein levels have also been shown to be significantly decreased in fibroblasts from patients possessing compound heterozygous mutations (Campeau et al. 2014b; Lozano et al. 2016). These particular data are limited to non-CNS cultured cells for a small proportion of TBC1D24 mutations, but they do suggest that the range of disease phenotypes may result from a variable loss of *TBC1D24* expression (Campeau et al. 2014b; Lozano et al. 2016). To establish whether the diversity of phenotypes observed can be explained by a particular threshold of TBC1D24 protein stability, it would be essential to carry out a more systematic study of protein expression in equivalent experimental samples from a full range of disease cases. Such a dataset will help to determine whether the majority of cases are defined by a loss-of-function mechanism, or whether gain-of-function modes of action need to be considered further. Significantly, it has been shown recently that siblings with the same compound heterozygous missense variants can present different disease severity and progression (Lozano et al. 2016). Although these particular individuals are non-identical twins, this finding does suggest that both genetic and non-genetic components determine the presentation of TBC1D24-associated diseases (Lozano et al. 2016). Thus, despite the obvious pleiotropic effects of this gene, these recent studies highlight the complexity in establishing a direct correlation between genotype and phenotype in TBC1D24-associated disorders (Balestrini et al. 2016).

### TBC1D24 in neuronal migration, differentiation and vesicle trafficking

Epilepsy is characterised by spontaneous neuronal activity that often arises from defects in neuronal development, migration or synaptic transmission (Bozzi et al. 2012); therefore, the role of TBC1D24 in these specific processes has been assessed both in vitro and in vivo. Over-expression of wild-type Tbc1d24 in mouse cortical or hippocampal neurons enhances greatly axonal outgrowth and branching, while expressing disease-causing amino-acid changes in either the TLDc or TBC domain of TBC1D24 negates this axon growth phenotype (Balestrini et al. 2016; Corbett et al. 2010; Falace et al. 2010; Milh et al. 2013). Importantly, knock-down of Tbc1d24 expression in rat primary cortical neurons also stunted neurite outgrowth (Falace et al. 2014); this apparent delay of neuronal maturation led to functional abnormalities in these knockdown neurons, including a reduced frequency and amplitude of excitatory postsynaptic currents (Falace et al. 2014). In addition, reducing Tbc1d24 levels in neuronal progenitor cells of embryonic rat brain resulted in a defective radial migration and morphological maturation of pyramidal neurons, with reduction of their arborisation and transition from multipolar to bipolar neurons (Falace et al. 2014). These data demonstrate a critical role for TBC1D24 in regulating neuronal differentiation and are crucial when dissecting the possible mechanisms underlying TBC1D24-associated epilepsy. Indeed, given that pathogenic mutations may result in reduced protein levels (Campeau et al. 2014b; Lozano et al. 2016; Milh et al. 2013), it is possible—although not yet demonstrated—that glutamatergic neurons in patients are not fully differentiated and are unable to stimulate the inhibitory interneurons, leading to an imbalance of the excitatory and inhibitory signals (Bozzi et al. 2012).

TBC1D24 has also been shown to play a key role in neurotransmission. Indeed, the TBC1D24 *C.elegans* homologue, C31H2.1, was identified originally in an RNAi screen for genes that modulate the release of neurotransmitter acetylcholine at the neuromuscular junctions (NMJ) (Sieburth et al. 2005). In a more recent screen for genes affecting synaptic transmission in *Drosophila*, an allelic series of mutants in the Skywalker (*sky*) TBC1D24 orthologue were characterised. A reduction in *sky* expression by more than 75% of control levels led to an increased number of synaptic vesicles and increased neurotransmission at the NMJ of *Drosophila* larvae (Uytterhoeven et al. 2011; Verstreken et al. 2009). This phenotype was linked to perturbed endo-lysosomal sorting, an overly efficient degradation of ubiquitinated proteins, and larger readily releasable vesicles; it was proposed that the resulting effects on neurotransmission might relate to aberrant neuronal activity observed in human seizures (Uytterhoeven et al. 2011).

In addition to the TLDc domain, TBC1D24 harbours a TBC domain, which is found in the majority of Rab-GTPase activating proteins (RabGAPs) (Frasa et al. 2012). Importantly, the TBC domain present in human TBC1D24 lacks the typical arginine ‘finger’ required for GAP activity and activation of Rab GTPases (Corbett et al. 2010; Fischer et al. 2016). Similar to the TBC proteins TRE17 and TBC1D3, that also lack the same critical arginine, TBC1D24 binds the small GTPase ADP ribosylation factor 6 (ARF6) (Frittoli et al. 2008; Martinu et al. 2004) and it inhibits the formation of active ARF6-GTP (Falace et al. 2014, 2010). ARF6 plays many important roles in synaptic vesicle trafficking (Fassio et al. 2016; Frittoli et al. 2008; Tagliatti et al. 2016), cell migration stimulation and cell guidance (Akiyama and Kanaho 2015; Santy and Casanova 2001), as well as neurite outgrowth and dendritic spine maturation (Albertinazzi et al. 2003; Choi et al. 2006; Miyazaki et al. 2005). As many TBC1D24 pathogenic variants identified in patients are located in the TBC domain, this suggests that TBC1D24 function may rely, at least partially, on an interaction with ARF6. Indeed, inactive ARF6-GDP rescued the radial migration defects observed *in vivo* that are associated with *Tbc1d24* knockdown (Falace et al. 2014), while electroporation of active ARF6-GTP phenocopied a reduction in *Tbc1d24* expression (Falace et al. 2014). Together, these data support the hypothesis that TBC1D24 plays a key role in synaptic transmission via regulation of ARF6 activation. It is likely that, in addition to ARF6, other GTPases are a feature of TBC1D24 function at the synapse. Indeed, it has been shown that TBC1D24 can also inactivate Rab35 (Uytterhoeven et al. 2011) and that Arf6 and Rab35 can inhibit each other by recruitment of reciprocal GAPs (Chaineau et al. 2013; Dutta and Donaldson 2015). Therefore, it is possible that the ratio of active to inactive forms of Rab35 and Arf6 are critical to regulate TBC1D24-associated synaptic function.

It is noteworthy that mouse hippocampal neurons lacking Arf6 demonstrate an accumulation of endosomal structures at the synapse upon stimulation, similar to the synaptic phenotype observed in *Drosophila* neurons with reduced *sky* levels (Tagliatti et al. 2016; Uytterhoeven et al. 2011). However, this apparently related synaptic phenotype was suggested to have arisen by different pathways in the two studies: loss of Arf6 leads to an apparent deficiency in vesicle formation upon stimulation (Tagliatti et al. 2016), while loss of *sky* leads to an increase in synaptic vesicle trafficking via the intermediate endosomal compartment and defect in vesicle recycling (Uytterhoeven et al. 2011). These experiments indicate that although TBC1D24 and ARF6 are able to modulate vesicle trafficking, their functional relationship in disease is more complex than simply the aberrant activation of ARF6 due to loss-of-function TBC1D24 mutations. Therefore, further studies are required to ascertain the in vivo functional significance of the TBC1D24:ARF6 interaction, particularly in the light of such a wide array of pathogenic TBC1D24 mutations documented to date.

While functional studies have focused on the role of TBC1D24 at the synapse, it must also be considered that TBC1D24 confers neuronal resistance against OS via its TLDc domain (Finelli et al. 2016); for example, epilepsy-associated mutations appear to be detrimental to OS resistance in neurons (Finelli et al. 2016). These data are also consistent with the modulation of an epileptic phenotype. Indeed, hallmarks of OS-dependent damage, such as lipid peroxidation of erythrocyte membrane, are observed in epileptic patients (Sudha et al. 2001). It has also been proposed that an increase in OS leads to synaptic dysfunction, neuronal hyperactivity and neuronal loss [reviewed in (Aguiar 2012; Chang and Yu 2010)]. Similarly, OS is proposed to play a central role in hearing loss, in particular age-related forms (Fujimoto and Yamasoba 2014); although it is unknown at present whether hearing loss-associated mutations affect the antioxidative function of TBC1D24. For example, as Tbc1d24 is expressed at a specific early postnatal time period (before P7) during development of the hair cells in the inner ear of the mouse, it will be important to investigate whether this confers protection against OS-associated insults (Azaiez et al. 2014; Rehman Atteeq et al. 2014; Zhang et al. 2014). In summary, the complex and pleiotropic effects of TBC1D24 will have to be considered when designing future therapeutic strategies for TBC1D24-associated disorders.

## Nuclear receptor coactivator 7 (NCOA7)

### NCOA7 is a nuclear receptor coregulator modulating gene transcription

NCOA7, originally identified as estrogen receptor-associated protein 140 (ERAP140) (Shao et al. 2002), contains a putative estrogen receptor-binding domain that had also been identified in ERAP160, a proven estrogen receptor alpha (ERα) interactor (Halachmi et al. 1994). Similarly, NCOA7 interacts with ERα and with an array of nuclear receptors such as ERβ1, ERβ2, the ligand-activated transcription factor arylhydrocarbon receptor (AhR) and its nuclear transporter (Arnt) (Arai et al. 2008; Cho et al. 2015; Paramanik and Thakur 2010), as well as other coregulators such as NCOA3 (Lanz et al. 2010). Binding of NCOA7 to its target nuclear receptors modulates their transcriptional activity, which suggests that NCOA7 acts as a transcriptional coregulator (Arai et al. 2008; Cho et al. 2015; Nguyen 1999; Shao et al. 2002). Nuclear receptor coregulators are essential rate-limiting intermediates between nuclear receptors and the general transcriptional machinery, influencing fundamental processes in the nervous system (Tetel and Acharya 2013). For example, Ncoa7 enhances the binding of the aryl hydrocarbon receptor (AhR/Arnt) complex to the dioxin response element of AhR/Arnt target genes under dioxin and estradiol exposure (Cho et al. 2015; Nguyen 1999; Xie et al. 2016). In addition, chromatin immunoprecipitation revealed that NCOA7, when bound to ERα or Ahr, associates with the promoter of their target genes, pS2 and cyclin D1, respectively, inducing their expression (Shao et al. 2002; Xie et al. 2016). Moreover, the expression of NCOA7 can be regulated by nuclear receptor ligands themselves; for instance, 1.0 nM androgen significantly reduces the expression of NCOA7 by 40% in prostate cancer cells (Heemers et al. 2009). However, the functional significance of NCOA7 to genome-wide transcriptional regulation is not known, in particular when compared to well-characterised nuclear coregulators such as steroid receptor coactivator 1 (SRC-1) (Walsh et al. 2012). Furthermore, as Ncoa7 can be highly protective against OS in vitro (Finelli et al. 2016; Yu et al. 2015), it is also possible that the protein regulates the expression of antioxidant enzymes, although this phenomenon is unlikely to involve the shortest isoforms as it lacks the nuclear localisation sequence or putative ER-binding domain of the full-length protein (Fig. 1).

### Additional potential functions of NCOA7

Whether NCOA7 plays a direct role in human disease is still unclear. However, NCOA7 expression is significantly induced by twofold in models of Rhinovirus infection (Baines et al. 2013), by almost threefold in bacterial lipopolysaccharides (LPS)-activated macrophages (Alasoo et al. 2015) or by twofold in LPS-activated monocytes (Schirmer 2008), which suggests a role for Ncoa7 in the immune response. In addition, NCOA7 has been consistently identified as highly induced by interferons (IFNs), which are potent cytokines used to treat a wide variety of diseases [reviewed in (González-Navajas et al. 2012)]. Treatment with an array of IFNs, for example IFNα in malignant melanoma (Zimmerer et al. 2008) or IFNα2b for chronic hepatitis C infection (Honda et al. 2010), induces the expression of NCOA7; although the specific isoforms involved in these studies were not reported. A more recent study has highlighted the importance of distinguishing these NCOA7 isoforms; in peripheral blood mononuclear cells from multiple sclerosis patients treated with IFNβ1b, a unique short NCOA7 isoform was significantly induced by more than 60-fold only 4 h post-IFNβ1b treatment (Yu et al. 2015). Interestingly, this specific isoform is induced by LPS but not OS, suggesting a specific role for this isoform as part of an immune response (Yu et al. 2015). In addition to its role in inflammation, IFN is implicated in the OS response via the transcriptional regulation of antioxidant genes (Croze et al. 2013; Lucas et al. 2003); thus, it is possible that NCOA7 may be a downstream regulator of IFN, directly or indirectly regulating the immune response and OS response.

### TBC/LysM-associated domain containing 1 (KIAA1609)

The *C.elegans eak-7* gene was first discovered as part of a screen for genes regulating DAF-16/FOXO, both key transcription factors promoting life span extension and stress resistance (Alam et al. 2010). Because *Eak7* encodes a protein with a consensus *N*-myristoylation motif and a TLDc domain, it was suggested to be a putative orthologue of KIAA1609 (Moriya et al. 2013; Williams et al. 2010). Eak-7 inhibits activity of the transcription factor Daf-16, ultimately reducing the expression of specific target genes (Alam et al. 2010). *Eak-7* null mutants showed an enhancement in the duration of the quiescent physiological larval stage and in their lifespan; this phenotype being reverted by neuronal-specific expression of eak-7 (Alam et al. 2010). The *N*-myristoylation domain in eak-7 facilitates its localisation to the plasma membrane; however, preventing *N*-myristoylation does not disrupt completely eak-7 function (Alam et al. 2010); thus, localisation to the membrane is not an absolute requirement.

The potential involvement of KIAA1609 in the OS response has also been tested; however, conflicting results have been observed in *C.elegans* and mammalian cells. Indeed, *eak-7* null mutants in *C.elegans* present an increased survival after exposure to H_2_O_2_ (Alam et al. 2010), while Kiaa1609 silencing in mammalian neuronal cell line treated with arsenite, an OS-induced agent, increases apoptosis (Finelli et al. 2016). Conversely, over-expressing Kiaa1609 in neurons was protective (Finelli et al. 2016). Thus, KIAA1609 is likely involved in the OS response, although its effect—protective or detrimental—might be organism and cell-type specific.

## TLDc proteins as therapeutic targets?

Given the importance of OS in the pathophysiology of neurodegenerative disorders (Melo et al. 2011), strategies employing antioxidants have been tested successfully in animal models of neurodegenerative diseases (Kim et al. 2015; Melo et al. 2011). However, clinical trials based on exogenous intake of low-molecular weight compounds with antioxidant properties have been only partially successful [reviewed in (Kim et al. 2015)]. For example, in early-stage PD, treatment with the monoamine oxidase inhibitor selegiline was able to delay the onset of major disability (Shoulson 1998), although it did not affect long-term symptoms nor extend life (Shoulson 1989, 1998). This limited success could be due, in part, to the fact that the small molecule antioxidants used reduce only one subtype of ROS. For example, coenzyme Q specifically targets superoxide radicals, but has no effect on the other ROS such as hydroxyl radicals, peroxides, H_2_O_2_ or hydroxyl ions (Phaniendra et al. 2015). Moreover, free radical scavenging alone is unlikely to reverse the established damage to DNA, proteins, lipids, and organelles that would have already occurred by the time of treatment (Amor et al. 2014).

Thus, to overcome these limitations, it has been suggested that activation of endogenous antioxidant proteins that target a range of pathways would be a better approach than the use of exogenous compounds (Chan and Chan 2015). In this context, the identification and characterisation of novel endogenous antioxidant proteins—such as those containing a TLDc domain—may be a valuable approach for the design new therapeutic strategies against neurodegeneration. Importantly, mice over-expressing Oxr1 in their CNS have normal phenotype (Liu et al. 2015), which demonstrates that increasing OXR1 levels would not be detrimental if used as part of a therapeutic strategy. As discussed above, over-expression of Oxr1 in vivo delays neurodegenerative phenotypes driven by a reduction in OS levels and inflammation (Liu et al. 2015) and intravenous delivery of stably transfected human OXR1 in MSCs resulted in a reduction of OS and inflammation in a mouse model of lupus nephritis (Li 2014). Thus, there is growing evidence that over-expression of OXR1 could be a viable and safe clinical approach.

If TLDc-containing proteins show promise as potential therapeutic targets, strategies aimed at identifying activity-modulating compounds are confounded by a limited knowledge of the TLDc domain itself. Although OXR1 appears to lack enzymatic activity, recent studies have described the importance of highly evolutionary conserved amino-acid residues in the TLDc domain that are required for antioxidant capabilities (Finelli et al. 2016). Importantly, to assist these studies, the three-dimensional structure of the TLDc domain of Ncoa7 from zebrafish has been solved (Blaise et al. 2012). These data revealed that the domain consists of four alpha-helices and ten beta-strands (Finelli et al. 2016), yet it contains no structural similarity to any known protein folds, providing no clear functional clues (Blaise et al. 2012). Most recently, the three-dimensional structure of the TBC domain from the TBC1D24 *Drosophila* orthologue *skywalker* (*sky*) has been described (Fischer et al. 2016). These data revealed a cationic binding pocket for phosphoinositide phosphorylated at positions 4 and 5 (PI(4,5)PI_2_ or PI(3,4,5)PI_3_) that appear to be structurally distinct from other known phosphoinositide binding domains (Fischer et al. 2016; Lewis et al. 2011). Furthermore, it was demonstrated that certain conserved residues mutated in DOORS syndrome prevented membrane association of the *sky* protein, generating the hypothesis that the resulting increase in vesicular trafficking influences synaptic transmission and causes the hyperactive phenotype observed in *sky* mutants (Czech 2000; Del Signore and Rodal 2016; Fischer et al. 2016). However, it is unknown whether this particular pathway is significant in disease pathogenesis; in particular, the *sky* mutants studied do not model all known DOORS syndrome patients, and overall they represent only a small proportion of all TBC1D24-associated mutations that occur throughout the protein sequence (Balestrini et al. 2016). Nevertheless, new structural observations such as these are an important step towards understanding why mutations in TBC1D24 cause such a range of serious neurological disorders. Furthermore, deciphering the common molecular mechanisms that underlie the function of the TLDc domain across the TLDc family will benefit research into TBC1D24-associated disorders, in addition to the potential application of OXR1 up-regulation as future therapeutic strategy.

## Future directions

Many questions regarding the functions of the TLDc family remain unanswered. Here, we have discussed the common neuroprotective properties of these proteins as conferred by the TLDc domain; however, it is unknown to what extent compensatory mechanisms occur when one TLDc protein is disrupted. As outlined above, specific isoforms of the TLDc proteins are present in distinct cellular compartments and can be induced by different cellular stimuli; although it is still unclear whether these proteins act via similar mechanisms under given cellular conditions. For example, could loss-of-function mutations in one TLDc protein be ‘rescued’ by another? Furthermore, are TLDc proteins independent of, or upstream of, well-known antioxidant pathways? Future studies are certainly required to shed more light on these important aspects of functional specificity, commonality and redundancy.

The clinical importance of TBC1D24—including mutations in the TLDc domain itself—is now well-established and there is an obvious need to understand the complex functional consequences of pathogenic mutations right across the protein structure. The pleiotropic effect of gene disruption is not an uncommon phenomenon in neurological disease (Valtorta et al. 2016), and initial mechanistic studies are pointing towards a key role for TBC1D24 in vesicle trafficking. Nonetheless, there is a need for a more systematic approach in model systems to help elucidate the intricacies of genotype and phenotype across the spectrum of TBC1D24-associated disease; in particular, as many more pathogenic mutations are likely to be described in the coming years as sequencing-based diagnoses becomes more routine.

The role of other TLDc proteins in human disease remains to be elucidated, although a very recent whole-exome sequencing study from families with specific language impairment (SLI) identified an inherited heterozygous mutation that introduces a premature stop codon in the first unique exon of the shortest OXR1 isoform (OXR1-C, Fig. 1) (Chen et al. 2017). These data are particularly intriguing, as it indicates that haploinsufficiency for a short TLDc domain-containing isoform leads to a neurodevelopmental phenotype. Not only does this finding suggest that the role of OXR1 in early development warrants further study, it provides further evidence that the subtleties of TLDc domain-containing protein function will extend to many, as-yet unexplored aspects of nervous system biology.
